# A Single‐Cell Atlas of Crab Ovary Provides New Insights Into Oogenesis in Crustaceans

**DOI:** 10.1002/advs.202409688

**Published:** 2024-11-18

**Authors:** Li Lu, Tao Wang, An Liu, Haihui Ye

**Affiliations:** ^1^ State Key Laboratory of Mariculture Breeding Fisheries College of Jimei University Xiamen Fujian 361021 China

**Keywords:** crustaceans, oogenesis, snRNA‐seq, transcription factors

## Abstract

Oogenesis is crucial for sexual reproduction and provides the material basis for population continuation. Nonetheless, the identity of the cells involved, the nature of transformation, and underlying regulators of oogenesis in crustaceans remain elusive. Here, an atlas of the ovary is plotted via single‐nuclei RNA sequencing (snRNA‐seq) in the mud crab *Scylla paramamosain*, resulting in five cell types, including germ cells, somatic cells, and three follicle cell types identified, which in turn provides abundant candidate markers for them. Moreover, profiles of ligand‐receptor in different cells of the crab ovary indicate the roles of cell communication in oogenesis. Dozens of transcription factors in the trajectory from oogonia to oocytes as well as the key molecules/pathways in somatic cells and follicle cells relevant to oogenesis are screened, which is evolutionarily conserved and its underlying regulatory mechanism is subject to some modification across various phyla. The spatiotemporal expression patterns of seven markers are further verified and the RNAi confirms the essential roles of piwi and VgR in oogenesis. These data help to elucidate the mechanism underlying gametogenesis and the evolution of reproductive strategy in invertebrates.

## Graphical abstract

Crabs are a valuable resource for aquatic ecosystems worldwide. This study presents a high‐resolution single‐cell atlas to study the oogenesis process in crabs. The results indicate the conserved trajectory of oogenesis from oogonia to oocytes. Moreover, based on the distribution features and subsequent functional analysis, the study determined several molecules that play decisive roles in crab oogenesis.

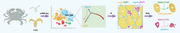

## 1 Introduction

Reproduction is a vital process for the survival and continuation of species.^[^ ^1^ ^]^ In the animal kingdom, amphigony contains several sequential events, including sexual differentiation, spermatogenesis/oogenesis, and mating.^[^ ^2^, ^3^, ^4^ ^]^ Oogenesis, in particular, is a well‐studied and programmed biological process that begins with primordial germ cells and progresses through the formation of oogonia and mitotic proliferation until sufficient primary oocytes are produced.^[^ ^5^ ^]^ These primary oocytes typically arrest at the prophase of the first meiotic division to accumulate nutrients, such as yolk proteins.^[^ ^6^ ^]^ Finally, the oocytes undergo subsequent meiotic division to reach maturation.^[^ ^7^, ^8^ ^]^

In crustaceans, oogenesis consists of four processes, germline cell proliferation, previtellogenesis, vitellogenesis, and oocyte maturation, which are defined by histological features.^[^ ^9^ ^]^ In brief, germline cell proliferation is the initial event in oogenesis, which typically commences with the mitotic division of oogonia. Several molecules have been identified to play pivotal roles during this process, including the ATP‐dependent RNA helicase DDX4 (*vasa*) and *piwi*, which are essential for the maintenance of germ stem cells, as well as germ cell specification and proliferation.^[^ ^10^, ^11^, ^12^ ^]^ Subsequently, the oocyte is distinguished by a pronounced surge in ribosomal gene transcription and the activity of the cytoplasmic organelles during the previtellogenesis stage.^[^ ^13^ ^]^ Vitellogenesis is an event during oogenesis, which is important for oocyte growth and contains three successive processes, including the first binding of vitellogenin (Vg) to its receptor VgR, followed by the endocytosis of the Vg/VgR complex, and the ultimate formation of yolk protein.^[^ ^14^ ^]^ Some molecules involved in this process have been identified, for instance, *Fem‐1*, *Erk2*, *Cdc2*, and *EGFR*.^[^ ^15^, ^16^, ^17^, ^18^, ^19^, ^20^ ^]^ However, most studies are limited to their molecular characterization and expression profiles. Ultimately, vitellogenesis terminates and the oocyte reaches the mature stage.^[^ ^13^ ^]^ It is also well established that crustacean oogenesis is regulated by many endocrine factors outside the ovary, such as the eyestalk neuropeptides (CHH, GIH/VIH, and MIH) and other hormones (methyl farnesoate, 20‐hydroxyecdyone, and bioamine).^[^ ^21^, ^22^, ^23^, ^24^ ^]^ Collectively, the interactions between different cell types in crustacean ovary are still not well understood, and the driving mechanism underlying oogenesis remains unclear.

Recently, single‐cell RNA sequencing (scRNA‐seq) or single‐nuclei RNA sequencing (snRNA‐seq) approaches have been utilized to investigate gametogenesis due to their high accuracy in detecting cellular variations and heterogeneity, as well as in predicting developmental trajectories.^[^ ^25^, ^26^ ^]^ For example, scRNA‐seq has been applied to construct molecular signatures of growing and regressing follicular populations in mammals, providing new insights into fertility by revealing reciprocal interactions between oocytes and somatic follicular compartments.^[^ ^27^ ^]^ In the Chinese tongue sole *Cynoglossus semilaevis*, several key transcription factors (TFs), including traf4, bcl11a, and sox11, are involved in germ cell differentiation.^[^ ^28^ ^]^ In fruit fly *Drosophila*, some markers have been reported, including *vasa*, *eyes absent* (*eya*), *Fasciclin 3*, *traffic jam* (*tj*), and *wnt4*.^[^ ^29^, ^30^, ^31^ ^]^ These identified markers based on a single‐cell atlas may be potentially informative for crustacean research. These studies revealed both remarkable interspecies variations and conservation during oogenesis across different phyla. However, scRNA‐seq analyses of gonadal cells in crustaceans have yet to be performed, although crustaceans represent an important taxonomic group found in aquatic ecosystems worldwide. Thus, it is necessary to precisely define the various cell types to gain a better understanding of the molecular mechanisms driving oogenesis.

In the present study, we investigated the oogenesis process in the mud crab *Scylla paramamosain*, a typical crustacean species widely distributed in the Indo‐Pacific region, which has great economic value due to its high nutritional benefits, especially the females with mature gonads.^[^ ^32^ ^]^ Eyestalk ablation has been employed to induce gonad maturation in crustacean industries.^[^ ^33^ ^]^ However, this method causes non‐negligible side effects, including permanent damage, poor gamete quality, and low fertilization rate.^[^ ^34^ ^]^ We aimed to explore the molecular mechanism involved in regulating oogenesis, thereby raising an opportunity to explore a new approach to meet this challenge. Using snRNA‐seq, we constructed an atlas of the ovary, determined the differentiation trajectory of germ cells, and revealed the cell communication in oogenesis. Moreover, we identified the expression profiles and developmental functions of several markers. Overall, these results provide valuable insights into crustacean breeding and enhance our understanding of the evolution of invertebrate reproductive strategies.

## 2 Results

### 2.1 Identification of Cell Types in the Ovary

**Figure 1:**
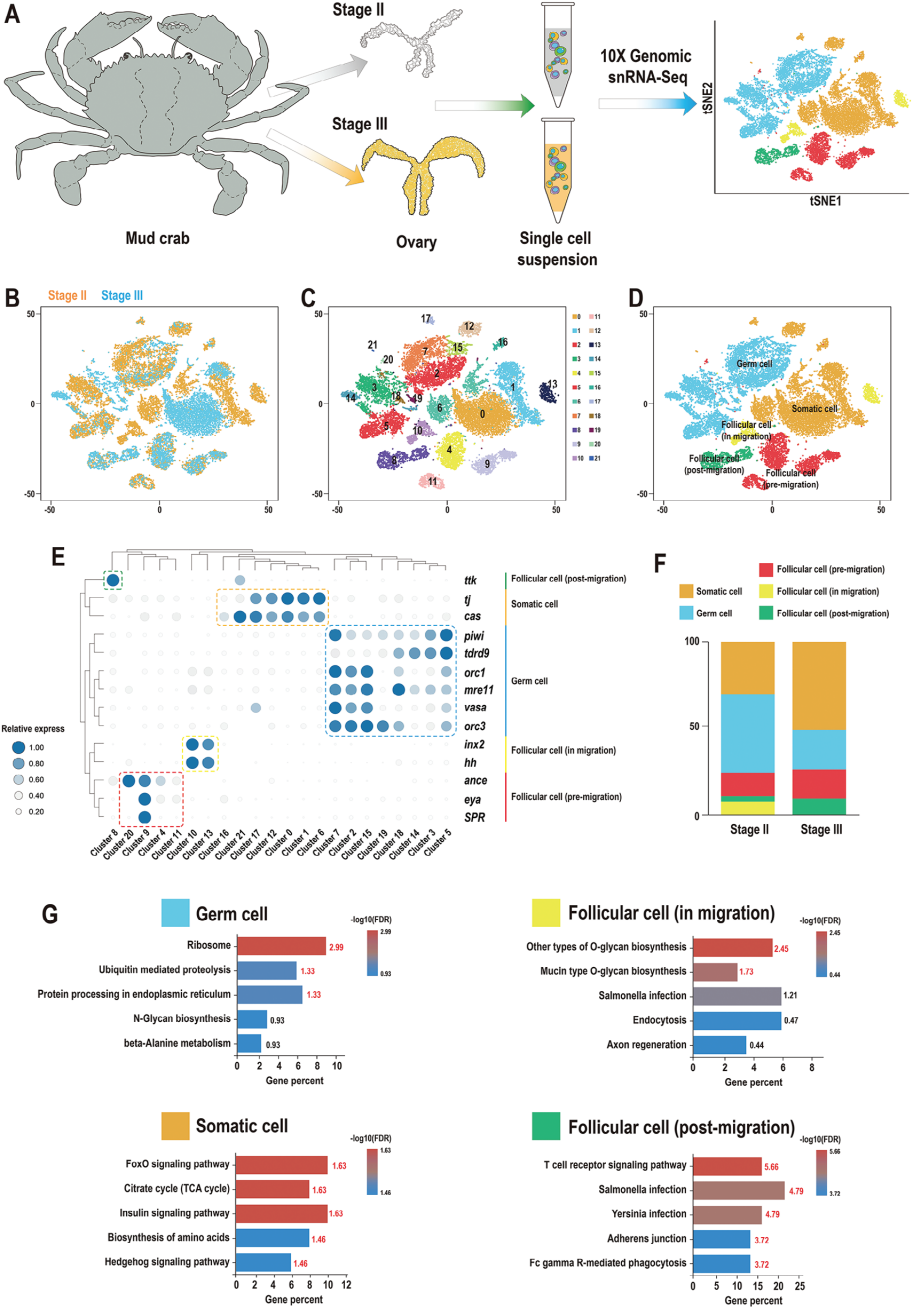
Overview of snRNA‐seq of the ovary of mud crab. A) Schematic representation of the snRNA‐seq analysis of mud crab ovary cells. B,C) The original classification of ovaries. D) Visualization of cell clusters using t‐SNE. Each point corresponds to a single cell and is color‐coded according to cluster membership. E) Heatmap of the normalized expression of marker genes in the five cell types. For each gene in each cluster, the expression levels are indicated by a color gradient; the percentage of cells expressing the gene is indicated by the size of the dot. Figure S2 (Supporting Information) shows the t‐SNE results for the marker genes. F) The proportion of each cell type. G) Enriched terms of DEGs are shown for ovary cell types (FDR values are shown).

The overall experimental workflow is depicted in **Figure** **1**A. We conducted an unbiased snRNA‐seq analysis on mud crab ovary at stages II and III since the vital transformation event of germ cells existed during these two stages. At stage II, the main germ cell type in the ovary was oogonia with a few primary oocytes. As the size of the ovary increased during stage III, the number of primary oocytes increased gradually and the cytoplasm appeared pink as the result of hematoxylin and eosin (HE) staining due to the abundance of yolk substances (Figure S1, Supporting Information). A total of 21539 cells were captured, with 13337 cells identified in the ovary at stage II and 8202 cells in the ovary at stage III. Additionally, the median number of genes and unique molecular identifiers (UMI) counts for these two samples were 1162 and 2322 at stage II and 990 and 2767 at stage III, respectively (Table S1, Supporting Information). These data were used for subsequent analyses.

Subsequently, 22 cell clusters (clusters 0–21) were generated and visualized on the t‐SNE plot (Figure 1B,C), leading to the identification of five cell populations including germ cells, somatic cells, and three follicle cell types based on expression profiles of well‐known markers (Figure 1D). Notably, eight clusters (2, 3, 5, 7, 14, 15, 18, and 19) were identified as germ cell populations expressing high levels of *vasa* and *piwi* markers. Seven clusters (0, 1, 6, 12, 16, 17, and 21) with high expression of somatic cell‐related genes, including *tj* and *castor*, were categorized as somatic cell populations. Furthermore, three types of follicular cells throughout the entire migration process were distinguished based on specific markers: four clusters (4, 9, 11, and 20) were classified as pre‐migration (*eya*), two clusters (10 and 13) as migration (*innexin 2* and *inx2*), and cluster 8 as post‐migration (*tramtrack* and *ttk*) (Figure 1E). The t‐SNE plot (Figure S2, Supporting Information) that describes the expression patterns of the aforementioned markers exhibits clear boundaries in each cell type, and the same cell type markers show a high level of overlap.

The changes in the cell population present in the datasets were assessed to examine the developmental trajectory. There was an increase in somatic cells proportion (30.35% to 50.84%) and a decrease in germ cells proportion (45.04% to 22.69%) from stage II to stage III (Figure 1F; Table S2, Supporting Information). There was little change in the overall proportion of follicular cells. Such a result was also consistent with the result of HE staining (Figure S1, Supporting Information). Subsequently, the differentially expressed genes (DEGs) in each population were analyzed. All DEGs in each population are listed in Data S2 (Supporting Information), and the heatmap revealed that the top 5 DEGs displayed clear separation boundaries from the remaining DEGs (Figure S3, Supporting Information). Furthermore, the KEGG annotations of these DEGs were analyzed and displayed in Figure 1G. In the germ cell population, the top five enriched processes were “ribosome”, “ubiquitin‐mediated proteolysis”, “protein processing in the endoplasmic reticulum”, “N‐glycan biosynthesis”, and “beta‐alanine metabolism”. In somatic cells, the significantly enriched pathways were the “Forkhead box O (FOXO) signaling pathway”, “citrate cycle (TCA cycle)”, and “insulin signaling pathway”. Additionally, “O‐glycan biosynthesis”, “mucin‐type O‐glycan biosynthesis”, and the “T‐cell receptor signaling pathway” were the chief enriched processes in follicle cells. However, no remarkable candidates were identified among the pre‐migration follicle cells.

### 2.2 Identification and Characterization of Germ Cell Subpopulations

**Figure 2:**
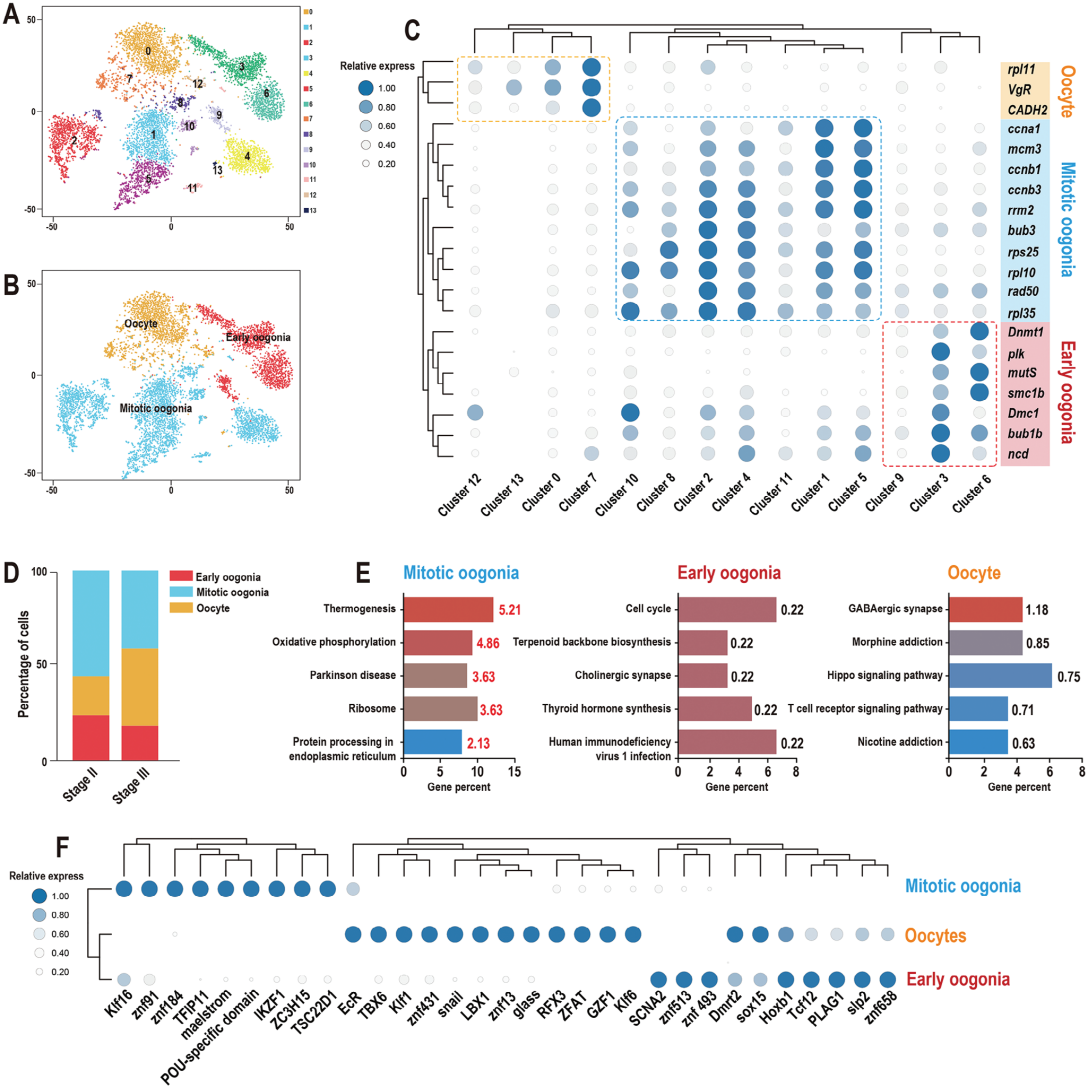
Gene expression dynamics and transcriptional characteristics of germ cells. A,B) t‐SNE plot of germ cell reclustering. C) Heatmap of the expression of marker genes in the three cell types. D) The proportion of each germ cell type. E) Enriched terms of sub‐germ cell DEGs (*p* values are shown). F) Heatmap of the expression of transcription factors in germ cells.

The germ cell populations were classified into a total of 14 subclusters (**Figure** **2**A) and subsequently clustered into three subpopulations: early oogonia, mitotic oogonia, and oocytes (Figure 2B,C). Specifically, subclusters 3, 6, and 9 were identified as early oogonia subpopulations due to their unique expression of landmarks, *mutS* and *dmc1*. Subclusters 1, 2, 4, 5, 8, 10, and 11 were considered to be mitotic oogonia subpopulations because they exhibited increased expression of mitotic genes (*mcm3*, *ccna1*, *ccnb1*, *ccnb3*, *rrm2*, *rps25*, *rpl10*, *rpl35*, and *bub3*). Subclusters 0, 7, 12, and 13 were categorized as oocyte subpopulations because they expressed the markers *VgR* and *rpl11*. The changes in the two germ cell samples were assessed, and the results indicated an increase in the proportion of oocytes and a reduction in the number of oogonia (Figure 2D), the results are consistent with HE in Figure S1 (Supporting Information).

A total of 3090 DEGs were identified among early oogonia, mitotic oogonia, and oocytes (Data S3, Supporting Information). Furthermore, the KEGG annotations of these DEGs were analyzed and displayed in Figure 2E, with “GABAergic synapse”, “Morphine addiction”, and “Hippo signaling pathway” enriched in oocytes. In the early oogonia population, “Cell cycle” and “Teroenoid backbone biosynthesis” were enriched. In mitotic oogonia, the enriched processes include “Thermogenesis”, “Ribosome”, and “Protein processing in the endoplasmic reticulum”. Additionally, the connections between these DEGs and known TFs were revealed and visualized through a heatmap (Figure 2F; Data S4, Supporting Information). Twelve TFs, including *EcR*, *TBX6*, *Klf1*, *znf431*, *snail*, *LBX1*, *znf13*, *glass*, *RFX3*, *ZFAT*, *GZF1* and *Klf6* were identified in the oocytes.

### 2.3 Developmental Trajectory of Germ Cells

**Figure 3:**
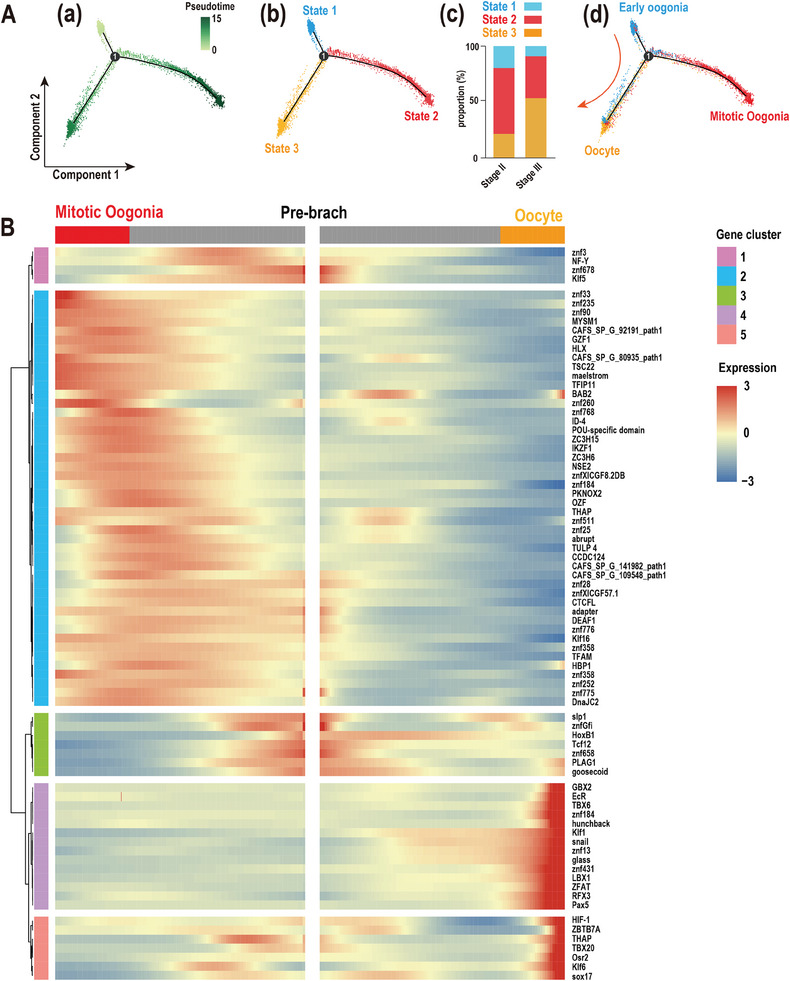
Developmental trajectory of germ cells. A) Trajectories of germ cells. (a,b) Distribution of the three trajectory states. c) Distribution of the three germ cell subsets in the trajectory. d) Cell numbers and percentages of each germ cell subset in the trajectory branches. B) Heatmap of the branch‐dependent transcription factors in the germ cell trajectory branch.

To gain insights into the fate of germ cells, we performed cell lineage reconstruction by positioning 7868 germ cells along a pseudotemporal axis. The developmental trajectory of the germ cell lineage was constructed and divided into three states by Monocle2 after assigning the origin. We observed three distinct states of germ cells at point 1 (**Figure** **3**A^[a&b]^). Subsequently, the developmental stage of the germ cells was determined, and their alterations were analyzed. There was an increase in cell proportion at state 3 and a decrease at states 1 and 2 in the crab ovary from stage II to stage III (Figure 3A^[c]^). It revealed that predominant early oogonia, mitotic oogonia, and oocytes were assigned to states 1, 2, and 3, respectively (Figure 3A^[d]^). Pseudotemporal analysis revealed a trajectory of gene expression associated with germ cell differentiation in cells at states 1, 2, and 3, providing us with the potential key TFs that regulate crab germ cell development (Figure 3B). This study identified a total of 77 TFs that were screened and clustered into five clusters. Notably, clusters 1 and 2 contained 50 TFs (such as znf3, NF‐Y, Klf5, and Klf16) that are potential regulators for inducing differentiation into mitotic oogonia, whereas clusters 4 and 5 included 21 TFs (GBX2, EcR, TBX6, etc.) that are involved in oocyte development.

### 2.4 Distribution of the Seven Selected Markers

**Figure 4:**
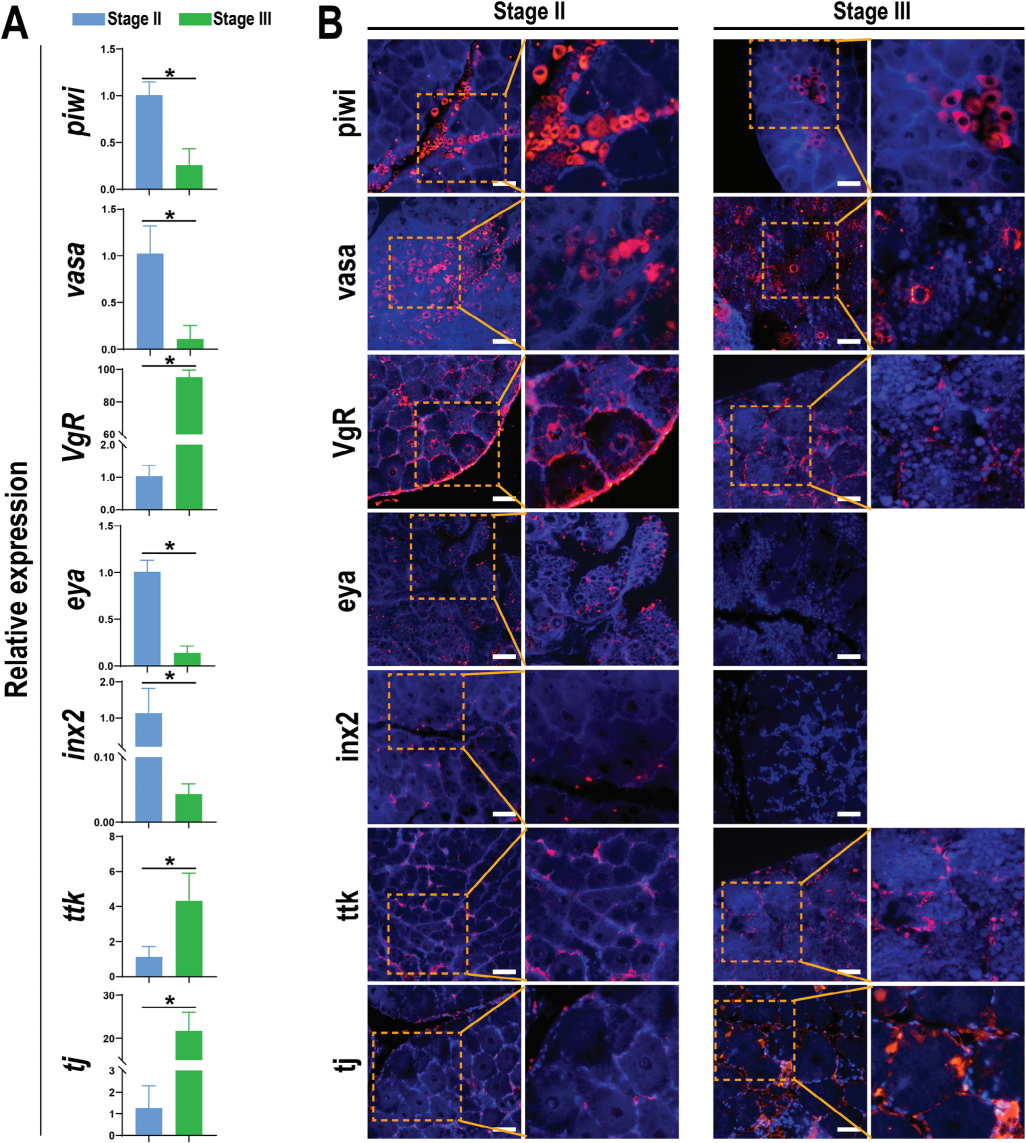
Spatiotemporal expression patterns of marker genes measured by qRT‒PCR and immunofluorescence. A) All values are expressed as the mean ± SD; n = 3. Asterisks indicate a significant difference (*p* < 0.05) between stages II and III. B,C) Locations of markers in mud crab ovary. The scale bars are 50 µm in length.

Seven markers, including *piwi*, *vasa*, *VgR*, *eya*, *inx2*, *ttk*, and *tj*, were screened based on their profiles via snRNA‐Seq, and the variation in transcript abundance was evaluated via qRT‐PCR (**Figure** **4**A). The results revealed that the abundances of *vasa*, *piwi*, *eya*, and *inx2* were significantly lower at stage III, whereas the abundances of *VgR*, *ttk*, and *tj* were upregulated during this period.

The specificity of the polyclonal antibodies against these molecules was verified through Western blot analysis. The results suggested that these antibodies can specifically bind to their antigen (Figure S4, Supporting Information), respectively, so they were employed for the subsequent experiments to describe the distribution of the markers in the ovary. Immunofluorescence staining revealed positive signals for vasa and piwi in the cytoplasm and nucleus of oogonia and primary oocytes, whereas VgR was localized in the cytomembrane of germ cells. Eya‐ and inx‐positive proteins were restricted to follicular cells at stage II, with pre‐migration and migration follicular cells detected, respectively. Notably, ttk‐positive cells surrounding the oocyte were observed among post‐migration follicular cells. Similarly, predominant tj‐positive cells surrounding the oocyte were observed at stage III (Figure 4B).

### 2.5 Roles of Piwi and VgR in Oogenesis

**Figure 5:**
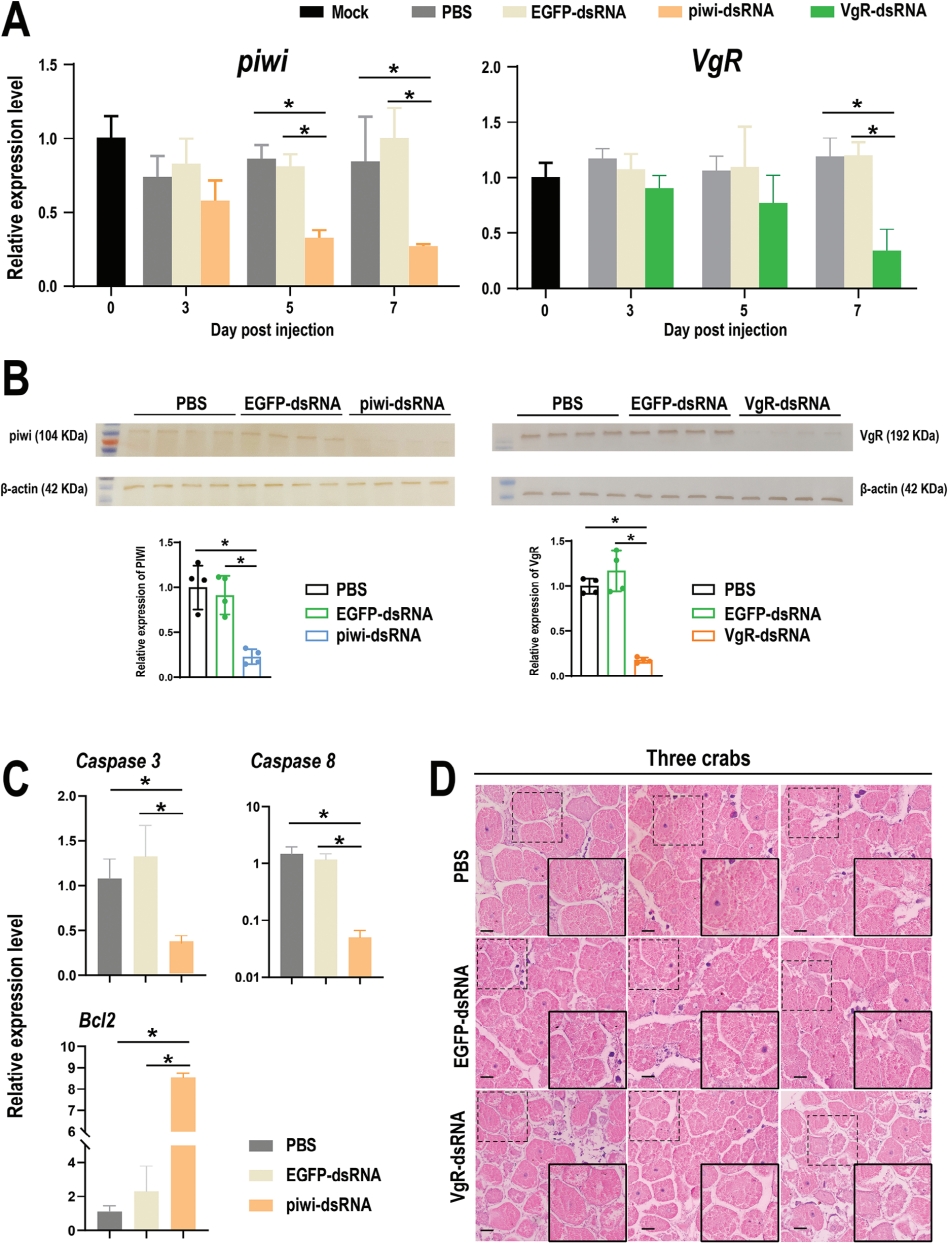
Influence of the oogenesis process after knockdown of piwi and VgR. A,B) Effects of dsRNA injection on piwi and VgR transcript levels and protein levels. Asterisks indicate a significant difference (*p* < 0.05). C) Effects of piwi gene knockdown on the transcription levels of apoptosis‐related genes. D) Effects of VgR gene knockdown on germ cell development. The scale bars are 50 µm in length.

The precise roles of two typical candidate genes (*piwi* and *VgR*) were further investigated through RNAi. The subsequent results indicated a significant knockdown of these two molecules at the transcript level on the 5th and 7th‐day post‐injection. Moreover, a remarkable reduction in the abundance of these two proteins was observed on the 7th day post‐injection (**Figure** **5**A,B). The *piwi* gene is associated with the process of cell death, especially apoptosis, and therefore the expression of the common landmarks *Caspase3*, *Caspase8*, and *Bcl2* were assessed. This analysis revealed that the transcript levels of the executors *Caspase3* and *Caspase8* were significantly suppressed, whereas the resister gene *Bcl2* was activated in the piwi‐dsRNA group (Figure 5C). Additionally, in the VgR‐dsRNA group, the histological results demonstrated an inhibition of oocyte development compared to that in the EGFP‐dsRNA group. This inhibition was characterized by a reduction in oocyte size and yolk accumulation in the oocytes (Figure 5D).

## 3 Discussion

Reproduction is a critical event in the lifespan of ovigerous animals. Traditional transcriptome analysis methods have been employed to study global gene expression patterns in ovaries. However, these methods do not account for cell‐to‐cell heterogeneity. With recent advancements in oogenesis research using scRNA‐seq technology, it is now possible to analyze gene expression characteristics at the single‐cell level through principal component analysis (PCA), overcoming these limitations. Inspired by these advancements, we utilized snRNA‐seq technology to construct a single‐cell atlas of crab ovary and investigated the potential mechanisms of oogenesis.

After standard quality control dataset filters, we observed lower UMI levels (≈2000) than in the fruit fly study (≈7000 UMIs per cell).^[^ ^29^, ^30^, ^31^ ^]^ This discrepancy may be due to the limited annotation of the crab genome. To address this issue, we established a third‐generation full‐length transcriptome library from the crab ovary and optimized the reference genome. This optimization resulted in a significant improvement in the mapping rate of the raw reads, with the mapping rate of the clean reads obtained from the stage II sample increasing to 38.3%, compared to the previous rate of 17.8% obtained using the previous genome. Furthermore, the optimized data allowed the identification of more cells and more marker genes. A similar strategy has been reported on the scRNA‐atlas of spleen tissue in bony fish which successfully characterized cell types without a reference genome.^[^ ^35^ ^]^ This study demonstrates the successful application of this strategy for the first time in crustaceans with more complex genomes and provides a reference for the optimization of single‐cell data in species with poorly annotated genomes. As a result, we initially identified five distinct cell groups, including germ cells, somatic cells, and three subpopulations of follicle cells, based on optimized data and well‐established markers. The t‐SNE plots illustrating the expression patterns of these candidates within each cell type demonstrated a high level of overlap and clear boundaries, emphasizing their conserved roles in crustacean oogenesis (Figure 1D).

Classical markers such as *vasa* and *piwi* were used to determine the ovary germ cell population of *S. paramamosain*.^[^ ^10^, ^11^, ^12^ ^]^ Consequently, three subtypes of germ cells (early oogonia, mitotic oogonia, and oocytes) were identified, and an increase in the proportion of oocytes was observed during oogenesis (Figure 1F). In total, more than 3000 highly expressed genes (Data S3, Supporting Information) were identified in these three cell types. Given their essential and conserved role in development, the TFs involved were further screened and analyzed (Data S4, Supporting Information). As a result, 31 TFs were identified, and their associations with different types of germ cells were revealed. For instance, the TF maelstrom is known to be crucial for the establishment of oocyte polarity during oogenesis in mice and fruit flies,^[^ ^36^, ^37^ ^]^ which are located primarily in the mitotic oogonia of crabs (Figure 2F). Additionally, *EcR*, a key downstream TF in the juvenile hormone and ecdysone signaling pathways necessary for female reproduction,^[^ ^2^ ^]^ was significantly expressed in crab oocytes. The finding suggests that *EcR* has the potential as a common marker for oocyte differentiation. Notably, certain TFs have not been previously reported to associate with oogenesis; for example, *LBX1*, a well‐known molecule involved in the migration of muscle precursor cells,^[^ ^38^ ^]^ requires further investigation. Subsequently, a dichotomous trajectory of germ cells originating from early oogonia was established. This analysis led to the identification of 7483 branch‐dependent genes, among which 78 TFs were clustered and displayed in a heatmap (Figure 3B; Data S5, Supporting Information). Significantly, 50 TFs, such as maelstrom and adapter, were positively correlated with the development of mitotic oogonia (Figure 3B). This observation aligns with previous results highlighting their distinct distribution in mitotic oogonia (Figure 2F). Besides, 21 TFs associated with oocyte development were obtained, and the majority of them were related to neurogenesis or body plan, such as *GBX2* and *TBX6* in zebrafish *Danio rerio* and frog *Xenopus laevis*.^[^ ^39^, ^40^ ^]^ Taken together, while the crustaceans and vertebrates belong to different reproductive models, the transcriptional regulatory network exhibits a certain degree of conservation and is subject to some modification across the gamogenesis taxa, exactly as Liu and his colleagues suggested.^[^ ^41^ ^]^

Additionally, we achieved the novel identification of somatic cells in the mud crab. Among the significantly enriched pathways observed in somatic cells, the well‐studied “FOXO signaling pathway and insulin signaling pathway” were prominent. FOXO, in general, plays a critical role in mediating the communication between insulin and JH signaling to coordinate insect development and reproduction.^[^ ^42^, ^43^ ^]^ Concurrently, insulin signaling acts cooperatively with gonadotropins to regulate diverse aspects of ovary development, including oocyte growth and maturation in vertebrates and invertebrates.^[^ ^44^ ^]^ Our result is consistent with the existing view that the insulin signaling pathway is highly conserved in vertebrates.^[^ ^44^ ^]^ However, the inconsistency of somatic cell markers between insects and crabs was also observed, such as the somatic cell marker *wnt4* in fruit fly^[^ ^29^ ^]^ was primarily detected in the germ cells of the crab ovary, whereas another somatic cell marker *mirr* in fruit fly^[^ ^29^ ^]^ was even absent in the crab genome. Moreover, the candidate markers for crab somatic cells also presented some evident differences compared to insects. For instance, *cas* was restricted to the somatic cells of mud crab, but it was well‐recorded in the follicular cells in fruit fly.^[^ ^30^ ^]^ Therefore, there are similarities in somatic cell behaviors among crabs and other metazoon, however, the variations among these phyla deserve attention.

A special population of somatic cells was identified as the primary mediator, referred to as follicle cells,^[^ ^6^, ^13^ ^]^ was also identified in this study. The expression of *eya*, *inx2*, and *ttk* was used to indicate the continuous developmental stages of follicular cells. Interestingly, *eya* and *inx2* were downregulated, whereas *ttk* was upregulated during this process (Figure 4A). Furthermore, a candidate marker of the post‐migration follicular cell, *wnt5b*, which is a member of the WNT family, was found to play a crucial role in the development of visceral organs in mammals and the movement of fish embryos.^[^ ^45^ ^]^ There have been reports of *wnt5b* linking to follicle cell development in the catfish *Clarias batrachus*, where *wnt5* is also present in the follicular layer.^[^ ^46^ ^]^ Therefore, it is likely that the involvement of *wnt5* and the wnt/β‐catenin signaling pathways in oogenesis is broadly shared among amphimictic populations. However, the well‐known somatic cell marker hedgehog^[^ ^29^ ^]^ was detected only in migration follicular cells in the mud crab, which suggests that the expression profiles and functions of specific genes are diverse among different phyla. Predictably, the core function of the follicle cell remains similar, but specific molecules and pathways undergo diversification during evolution.

Interactions between germ cells and somatic cells are pivotal for oogenesis,^[^ ^8^, ^31^, ^47^ ^]^ but the knowledge of relevant interplays in crustaceans remains limited. In this study, cell communication was also found during the crab oogenesis. Such as the *TGF‐βI receptor* (*TGFβRI*), the molecules belonging to the TGF‐β signaling pathway, mainly expressed in the post‐migration follicle cells. However, the ligand BMP7 is primarily detected in germ cells (Figure S5, Supporting Information), suggesting that bidirectional communications exist between germ cells and follicle cells, similar to the results in fish.^[^ ^28^ ^]^ Notably, the insulin receptor is mainly expressed in somatic cells, whereas *insulin‐like growth factor 2 mRNA‐binding protein 1* (*IGF2BP1*) is mainly expressed in germ cells (Figure S5, Supporting Information), suggesting that these functional genes may concert germline‐somatic interactions. Similar to the observations in fruit fly,^[^ ^46^ ^]^ a nucleus‐localized positive signal of tj was detected in the somatic cells adjacent to germ cells in the crab ovary (Figure 4B). Furthermore, the intensity of tj increased concomitantly with the developmental process. In fruit fly, *tj* undergoes migration during the rearrangement of cell types in the larval ovary, and is essential for determining niche cell fates and architecture. In mud crab, *tj* and *VgR* have high synchronous expression, which suggests that *tj*‐enriched somatic cells are necessary for the differentiation and development of gem cells. These observations underscore that cell communication exists between germ cells and somatic cells, which may coordinately regulate crustacean oogenesis and warrants future study.

*Piwi* was first identified in the germline stem cells of *Drosophila*, which have a role in maintaining germline stem cells, and the mutation of piwi stopped the division of germline stem cells.^[^ ^47^ ^]^ *VgR* plays an important role in egg yolk protein uptake at vitellogenesis.^[^ ^48^ ^]^ These two genes were also identified as markers of oogonia and oocytes in this study, respectively. To further elucidate the roles of *piwi* and *VgR* in crab oogenesis, an RNAi assay was conducted. Notably, in the Chinese mitten crab, *Eriocheir sinensis*, the apoptotic process was strongly influenced by the piwi protein in the testes.^[^ ^49^ ^]^ In *piwi* knockdown mud crabs, the repression of two apoptotic biomarkers was observed in the ovary. Similarly, piwi has been shown to regulate the apoptosis process in mouse ovary, whereas subsequent developmental processes of zygotes are well established.^[^ ^50^ ^]^ In the *VgR* knockdown group, alterations in germ cell location and a significant increase in ovary tightness were observed, consistent with the findings in insects such as the tropical warehouse moth *Cadra cautella*, further emphasizing their essential nature.^[^ ^51^ ^]^ In the present study, *piwi* and *VgR* were first identified as the markers of oogonia and oocytes at the cell‐specific resolution in crustaceans, respectively. Especially, the function of *piwi* during oogenesis in female crustaceans should be attached to importance as it plays a vital role in males. Many involved molecules were also screened, which provided new insights into the driving mechanism of oogenesis in crustaceans. Here, our RNAi results support the significance of these genes in oogenesis and offer an approach to further research on their precise roles.

In summary, our study reported the first comprehensive single‐cell transcriptomic atlas of crustacean ovary, identified five distinct cell populations of crab ovary, and characterized the candidate markers related to each cell type. This study enriched the markers in non‐model invertebrate species and enhanced the cell‐specific resolution to bulk gene expression studies. Furthermore, the trajectories of germ cells and the involved TFs as well as the transcriptional regulatory network of somatic cells/follicle cells, jointly shed new insights into the underlying molecular mechanism of oogenesis, which is evolutionarily conserved and subject to some modification across phyla. The cell communications between different cell types in the ovary were also revealed, which may regulate crustacean oogenesis and warrants future study. Moreover, the requirement of two crucial factors in oogenesis was determined, which provides a practicable model for function analysis of other interesting candidates. These findings contribute to a comprehensive understanding of reproductive strategies in invertebrates and provide essential data for research on reproductive biology.

## 4 Experimental Section

### Ethics Statement

All animal experiments in this study were approved by the Institutional Animal Care and Use Committee of the Fisheries College of Jimei University (Approval Code: 2021‐04; Approval Date: January 22, 2021).

### Animal and Tissue Preparation

The ovarian development of *S. paramamosain* has been divided into five stages in a previous study: stage I (undeveloped), stage II (previtellogenesis), stage III (early vitellogenesis), stage IV (late vitellogenesis), and stage V (mature), based on morphological and histological characteristics.^[^ ^52^, ^53^ ^]^ Female crabs at stage II (109.85 ± 19.57 g) and stage III (352.42 ± 46.88 g) were obtained from a local aquafarm in Xiamen City, China, and were cultured in filtered seawater for 15 days prior to subsequent experiments. The seawater temperature and salinity were maintained at 26 ± 0.5 °C and 27 ± 0.5 ppm, respectively. During this period, the crabs were fed live Manila clams (*Ruditapes philippinaru*m) on a daily basis.

The crabs were anesthetized using hypothermia. Two ovary samples, consisting of an equal mixture of three crab ovaries at stages II (white) and III (yellow) (Figure S1, Supporting Information), were collected for snRNA‐seq, full‐length RNA‐seq, total RNA extraction, and protein isolation (Figure 1A). Additionally, samples fixed in 4% paraformaldehyde (Solarbio, Beijing, China) were subjected to further experiments, including HE and immunofluorescence staining.

### RNA Isolation, cDNA Synthesis, and Real‐Time Quantitative PCR (qRT‐PCR)

Total RNA was extracted using TRIzol reagent (Invitrogen, Carlsbad, CA, USA, 15596026) and reverse‐transcribed using the PrimeScript RT reagent kit with gDNA Eraser from TaKaRa (Dalian, China). qRT‒PCR was performed using TB Green Premix Ex Taq II (Tli RNaseH Plus) (TaKaRa, Dalian, China) on a QuantStudio 5 Flex Real‒Time PCR System from Thermo Fisher Scientific (Waltham, USA). Reference gene *β‐actin* and 2^−ΔΔCt^ methods were used for normalization.

### Hematoxylin and Eosin Staining

The ovary samples were dehydrated using graded alcohol solutions (70%, 85%, 95%, and 100%), cleared in xylene, and embedded in paraffin wax. Subsequently, serial slices with a thickness of 7 µm, were stained using an HE staining kit from Beyotime (Shanghai, China, C0105S).

### SnRNA‐Seq Library Preparation and Sequencing

The crabs at stages II and III were collected and the corresponding single‐cell samples from the ovary were separated for library building and sequencing by Genedenovo Biotechnology Co., Ltd. (Guangzhou, China), based on the 10× Genomics platform (10× Genomics, Pleasanton, CA, USA). In brief, the single nuclear suspensions were prepared, and 3′ cDNA libraries were generated and sequenced using the Chromium Next GEM Single Cell 3′ Reagent Kit v3.1 and Illumina HiSeq 4000 instrument, respectively. Subsequently, the quality of raw data was assessed by Cell Ranger (v3.1.0), followed by the filtration of reads with low‐quality barcodes and UMIs and alignment to the optimized reference genome (Data S1, Supporting Information). UMI sequences for sequencing errors were corrected and the valid barcodes were identified using the EmptyDrops method, resulting in the cell‐by‐gene matrices produced through UMI counting and cell barcode calling. Seurat (v3.1.1) was employed to analyze the gene matrices of each sample since the Doublets were removed using DoubletFinder (v2.0.3), and cells meeting the following criteria were screened for downstream analysis: gene counts of 270–3700 per cell, UMIs with counts < 1300 per cell, and cells with a percentage of mitochondrial genes per cell < 10%.

### Cell Clustering and Cell type Identification

After retaining high‐quality cells, the matrix was normalized following the “log homogenization” method and then the data merging was performed through the Harmony algorithm. Next, PCA was conducted to select the best principal component for subsequent analysis, and the Euclidean distance between cells was estimated. Seurat was used to embed cells in an SNN (shared nearest neighbor) graph based on the Euclidean distance, and the resulting SNN graph was partitioned into highly interconnected quasipopulations based on the Jaccard distance between two cells in local proximity. After the clustering using Louvain's algorithm, the expression data of cells were visualized using nonlinear dimensionality reduction (t‐SNE). This process led to the identification of 22 initial cell clusters. Subsequently, common marker genes related to humans, teleosts,^[^ ^28^, ^38^ ^]^ and fruit flies^[^ ^29^, ^30^, ^31^ ^]^ were used to identify subgroups within the ovary.

### Gene Function Enrichment Analysis

Scanpy's rank sum test was utilized to screen genes with upregulated expression in each cell cluster, following the criteria that |logFC| > 1, *p* < 0.05, and gene expressed in more than 25% of the cells in the target cluster. For DEGs between the two groups in each cell type, the screening criteria were |logFC| > 1 and *p* < 0.05. The mapping of given genes to gene ontology (GO) terms or Kyoto Encyclopedia of Genes and Genomes (KEGG) pathways in the GO database (http://www.geneontology.org/; accessed on May 8, 2022) and KEGG database (https://www.kegg.jp/; accessed on May 8, 2022) was carried out through OmicShare software, with the p values were FDR‐corrected using a threshold of FDR ≤ 0.05.

### Cell Trajectory Analysis

To describe the process of oogenesis, the pseudotime trajectories were constructed using the Monocle 2 package (version 2.14.0; http://cole‐trapnell‐lab.github.io/monocle‐release/docs/) since the Seurat dataset was transformed into the cell_data_set. The ordered genes were selected to define cell progression. DDRTree was utilized to reduce the space to two dimensions, and all cells were ordered using the orderCells function with the default parameters.

### Antibody Preparation and Western Blot

To further evaluate the expression patterns of the candidate markers, polyclonal antibodies against seven marker proteins (piwi, vasa, VgR, eya, inx2, ttk, and tj) were prepared. Initially, the high‐efficiency epitopes of these seven proteins were assessed, after which the selected peptides (Table S3, Supporting Information) were synthesized. These peptides were coupled with keyhole limpet hemocyanin (KLH), mixed with Freund's complete adjuvant, and subcutaneously injected into New Zealand White rabbits. Polyclonal antibodies were subsequently extracted and enriched from the rabbit serum following secondary immunization with a mixture containing KLH peptides and Freund's incomplete adjuvant.

Total proteins from ovarian samples were isolated using a protein extraction kit from Solarbio (Beijing, China, BC3710). Each protein sample (20 mg) was subjected to 12% SDS‐PAGE and transferred to a nitrocellulose membrane (Beyotime, Shanghai, China, FFN02). The samples were blocked and incubated with primary antibodies diluted at a ratio of 1:1000 for 1 h. Subsequently, the membranes were washed three times in Tris‐buffered saline with tween (TBST, TBS+0.1% Tween‐20) and incubated with the secondary antibody HRP‐labeled goat anti‐rabbit IgG (H+L) (Beyotime, Shanghai, China, A0208) for 30 min. Finally, all the membranes were washed three times in TBST and the antigen‐antibody complexes were detected using a DAB horseradish peroxidase color development kit from Beyotime (Shanghai, China, P0203).

### Immunofluorescence

The ovary tissue samples were rehydrated as mentioned above. An EDTA antigen retrieval solution from Sangon Biotech (Shanghai, China, E673004) was then applied to retrieve the antigens. Afterward, the samples were blocked and the primary antibodies (vasa, piwi, VgR, eya, inx2, ttk, and tj) were diluted at a ratio of 1:100 and added to the samples, which were then incubated for 1.5 h. Afterward, the samples were washed three times with TBST and incubated with the secondary antibody Alexa Fluor 647‐labeled goat anti‐rabbit IgG (H+L) (Beyotime, Shanghai, China, A0468) for 1 h. Finally, the samples were observed since the incubation with 4‐6‐diamidino‐2‐phenylindole (DAPI) to label cell nuclei.

### RNA Interference

Double‐stranded RNA (dsRNA) was synthesized to investigate the in vivo function of *piwi* and *VgR*. Primers with a T7 promoter sequence, namely, dspiwi‐F/R and dsVgR‐F/R, were designed based on the cDNA sequence using the BLOCK‐iT RNAi Designer (https://rnaidesigner.thermofisher.com/rnaiexpress/). Enhanced green fluorescent protein (EGFP) was used as an exogenous control (dsEGFP). All primers used were listed in Table S4 (Supporting Information). A MEGAscript RNAi Kit (Thermo Fisher Scientific, USA, AM1626) was used to synthesize the dsRNAs. The dsRNA was extracted using phenol/chloroform, precipitated with isopropanol, washed with 80% ethanol, dried, and then dissolved in RNase‐free dH_2_O.

A total of 45 crabs at stage II were collected and randomly divided into three groups, with 15 crabs (n = 15) per group. In the PBS (phosphate buffer solution) group, 100 µL of PBS was injected. In the EGFP group, EGFP‐dsRNA was injected at a rate of 2 µg g^−1^ body weight and dissolved in 100 µL of PBS. In the piwi group, piwi‐dsRNAs were injected using the same protocol. Injections were performed using a microsyringe (Hamilton, Bonaduz, Switzerland) with a thin needle inserted into the arthrodial membrane at the fifth swimming leg. For the VgR silencing experiment, another 45 crabs at stage III were collected, and the injection regime was the same as that in the piwi silencing experiment, with VgR‐dsRNA being used instead of piwi‐dsRNA. Every three crabs (n = 3) were collected and sacrificed at four sampling points (3, 5, 7, and 14 days postinjection). Additionally, three crabs were collected on the 0th day as pre‐injection controls. Finally, the ovarian samples (3rd, 5th, and 7th day) were subjected to gene expression analysis by qPCR, protein analysis by Western blot, and histological analysis of the ovaries sampled on the 14th day postinjection via H&E staining.

### Drawings and Statistical Analysis

Adobe Photoshop CC (San Jose, CA, USA) and Adobe Illustrator (San Jose, CA, USA) were utilized for drawing and designing the final panel. All the data were presented as the means ± standard deviations (SD). Prism software (version 8.0) was used for fitting the regression equation, the one‐way ANOVA with post hoc tests (Tukey HSD test) and Student's t test was used to analyze significant differences. Asterisks indicated statistically significant differences (*p* < 0.05).

### Ethics Approval Statement

All animal experiments in this study were approved by the Institutional Animal Care and Use Committee of the Fisheries College of Jimei University (Approval Code: 2021‐04; Approval Date: January 22, 2021).

### Data Availability

The single‐cell RNA sequencing data generated in this study have been deposited in the NCBI Sequence Read Archive under accession number “PRJNA1118234”.

## Conflict of Interest

The authors declare no conflict of interest.

## Author Contributions

H.H.Y., A.L conceived and designed the experiments; L.L, TW performed the experiments; A.L, L.L analyzed the results; L.L wrote the manuscript; all authors revised the manuscript. All authors read and approved the final manuscript.

## Supporting information



Supporting Information

Supplemental Data 2

Supplemental Data 3

Supplemental Data 4

Supplemental Data 5

## Data Availability

The data that support the findings of this study are available from the corresponding author upon reasonable request.
