# Supplementary material for: A Single‐Cell Atlas of Crab Ovary Provides New Insights Into Oogenesis in Crustaceans
Source: Adv Sci (Weinh). 2024 Nov 18;12(2):2409688. doi: 10.1002/advs.202409688 (PMC11727118; doi:10.1002/advs.202409688)
Supplement: Supplementary file 1 — Supporting Information [file ADVS-12-2409688-s003.docx]

Supporting Information

**A Single-Cell Atlas of Crab Ovary Provides New Insights into Oogenesis in Crustaceans**

*Li Lu, Tao Wang, An Liu*, and Haihui Ye**

**This additional file includes:**

Supplemental Figures S1‑5.

Supplemental Tables S1‑4.

Supplemental Data S1.


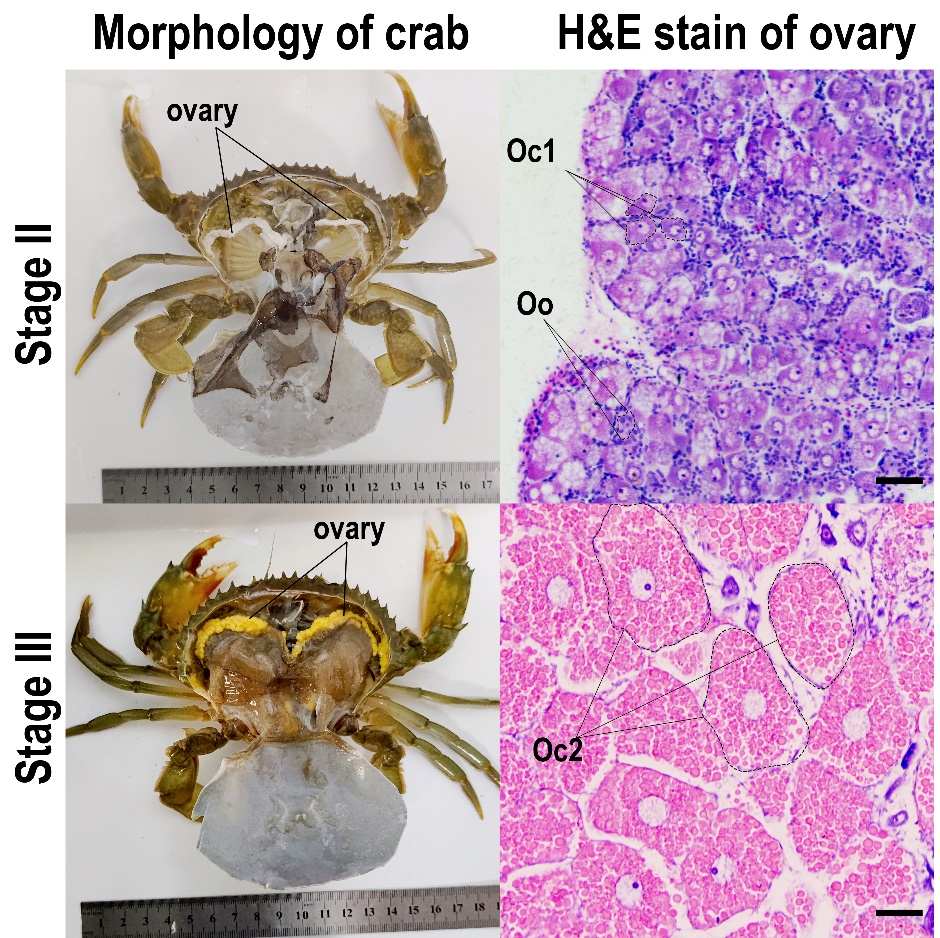


**Figure S1. Morphology and histology of mud crab ovary in stage Ⅱ and stage Ⅲ.** The scale bars are 50 µm in length. Oc1: previtellogenesis oocytes; Oc2: vitellogenesis oocytes.


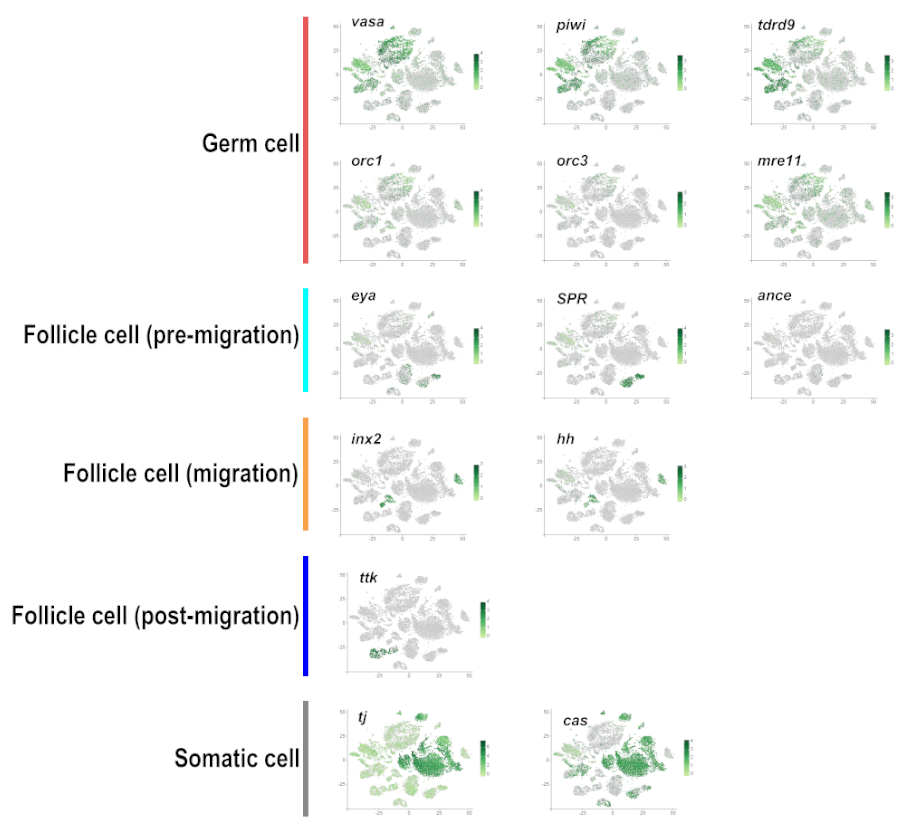


**Figure S2. t-SNE plots visualizing expression specificity of the marker genes for each cell type.**


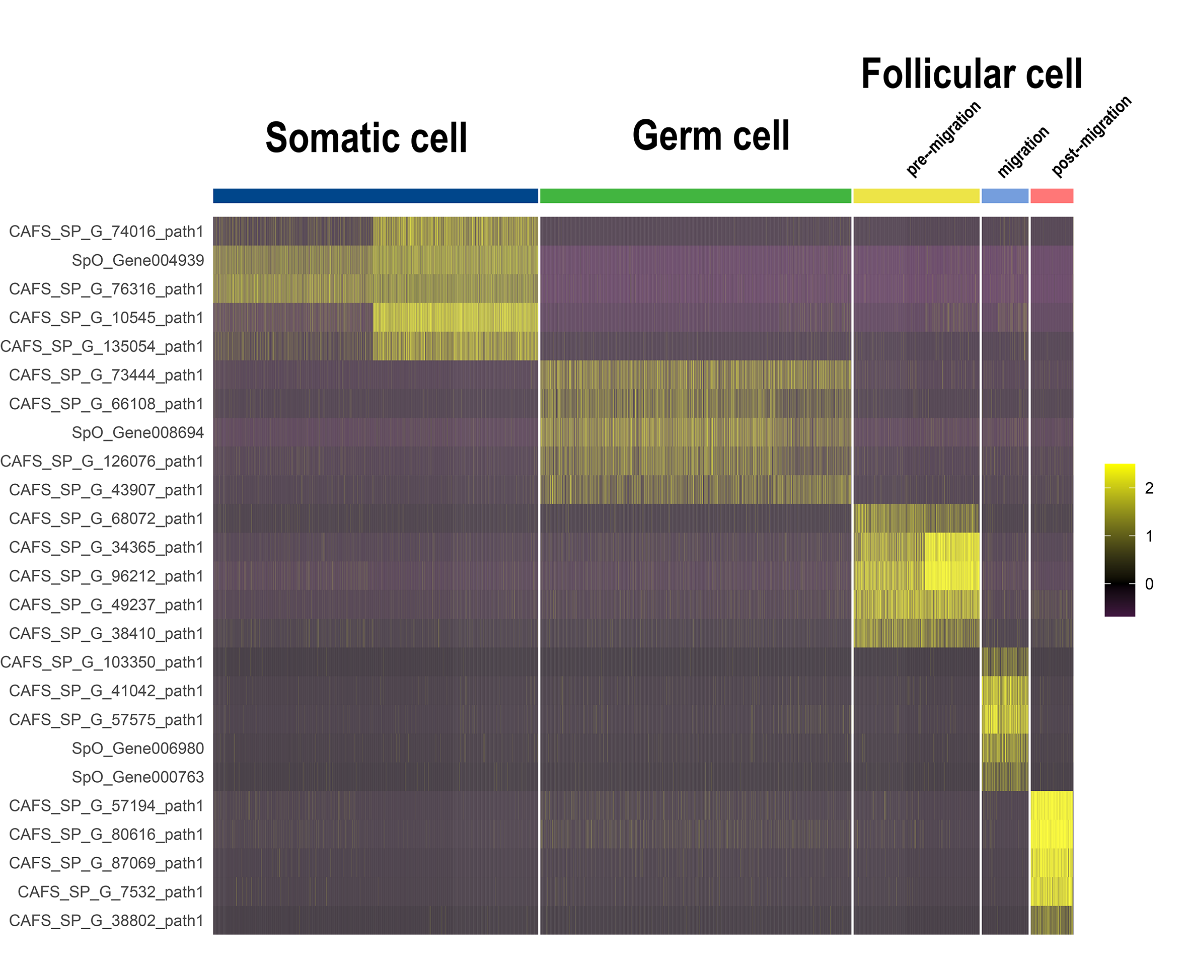


**Figure S3. Heat map exhibiting distinct expression patterning of the top 5 genes for each cell type.** Gene expression levels from low to high are indicated by a color gradient from purple to yellow.


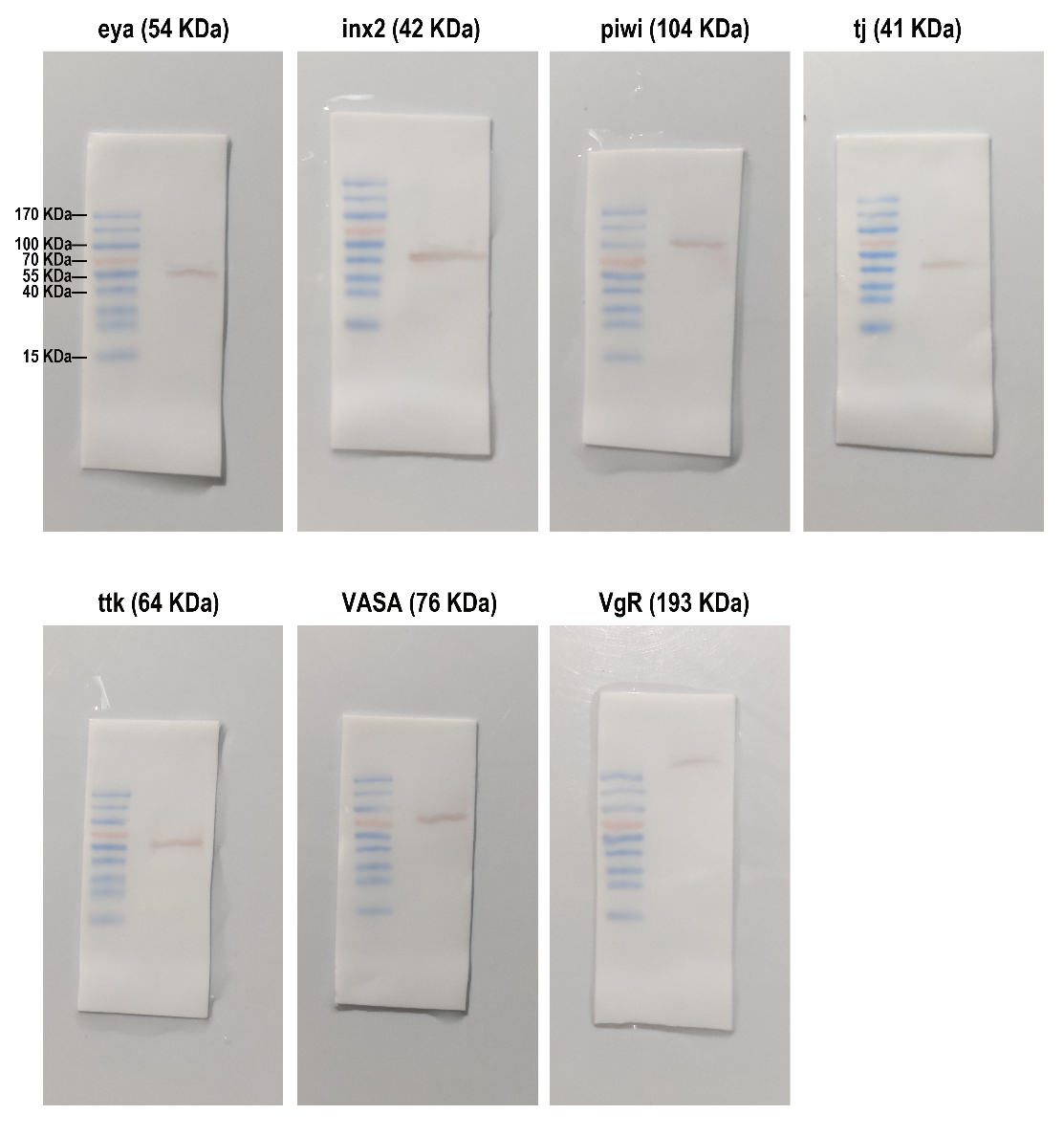


**Figure S4. Western blot results of markers.**


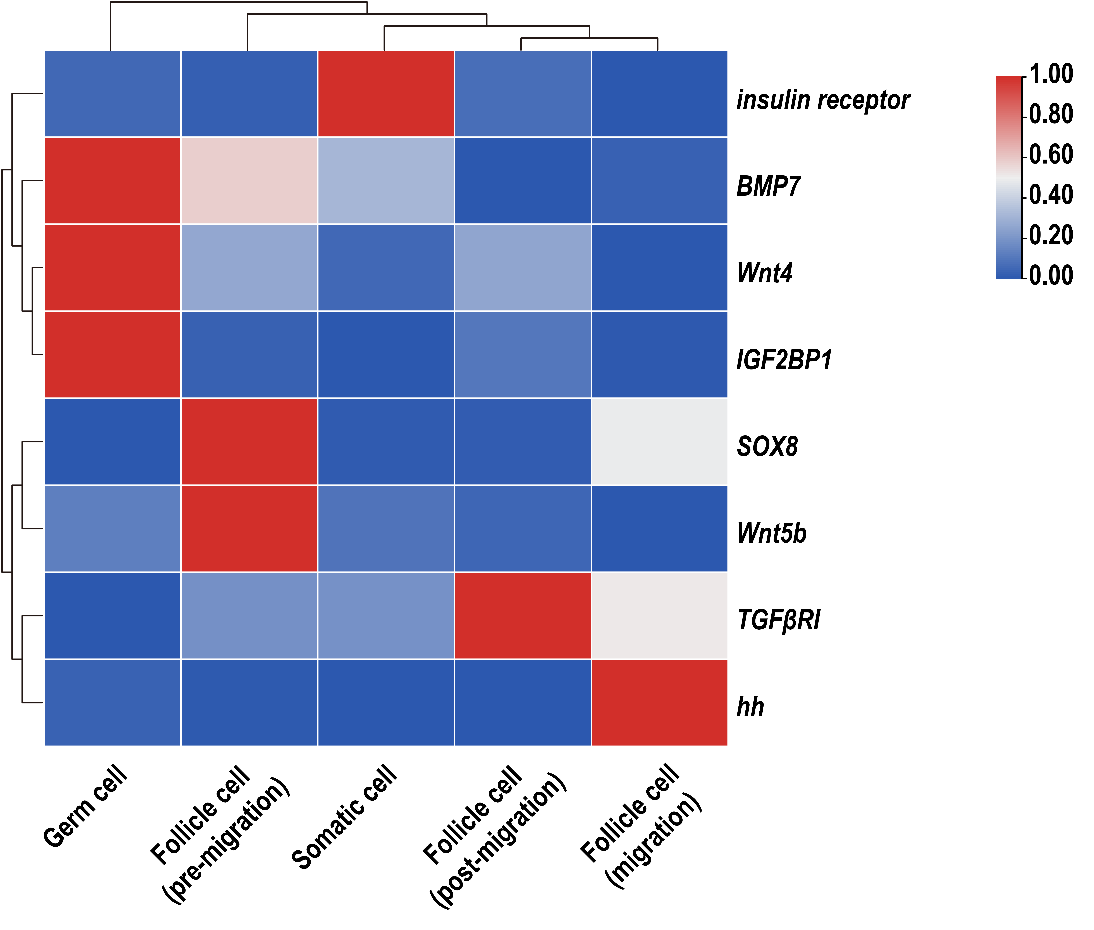


**Figure S5. Heat map exhibiting genes for each cell type.** Gene expression levels from low to high are indicated by a color gradient from blue and grey to red.

**Table S1. Base data of ovary scRNA-Seq.**

| Sample | Stage II | Stage III |
| --- | --- | --- |
| Number of Reads | 438,552,339 | 389,128,709 |
| Valid Barcodes | 96.20% | 96.90% |
| Sequencing Saturation | 48.90% | 46.90% |
| Q30 Bases in Barcode | 95.80% | 95.50% |
| Q30 Bases in RNA Read | 88.20% | 86.40% |
| Q30 Bases in UMI | 92.40% | 91.90% |
| Estimated Number of Cells | 15,257 | 9,130 |
| Fraction Reads in Cells | 85.40% | 68.40% |
| Mean Reads per Cell | 28,744 | 42,621 |
| Median Genes per Cell | 1,290 | 1,026 |
| Total Genes Detected | 17,272 | 17,027 |
| Median UMI Counts per Cell | 2,683 | 2,935 |
| Reads Mapped Confidently to Genome | 68.50% | 69.90% |
| Reads Mapped Confidently to Intergenic Regions | 13.00% | 13.10% |
| Reads Mapped Confidently to Intronic Regions | 18.80% | 18.30% |
| Reads Mapped Confidently to Exonic Regions | 27.40% | 25.60% |
| Reads Mapped Confidently to Transcriptome | 38.30% | 35.00% |
| Cell numbers after filter | 13,337 | 8,202 |
| Median UMI per cell after filter | 2,322 | 2,767 |
| Median genes per cell after filter | 1,162 | 990 |

**Table S2. Proportion of five cell types in ovary.**

| Cluster | Stage II | Stage III |
| --- | --- | --- |
| Somatic cell | 4048 (30.35%) | 4170 (50.84%) |
| Germ cell | 6007 (45.04%) | 1861 (22.69%) |
| Follicular cell (pre-migration) | 1812 (13.59%) | 1376 (16.78%) |
| Follicular cell (post-migration) | 407 (3.05%) | 777 (9.47%) |
| Follicular cell (migration) | 1063 (7.97%) | 18 (0.22%) |

**Table S3. Selection epitopes of seven markers in this research.**

| Name | Sequence |
| --- | --- |
| eya | STRSLNQSSKGALGSGS-Cys |
|  | DSPSSPLKDTASRAR-Cys |
| inx2 | KNYIGENIRCITGFEKQEHKAIE |
|  | Cys-LNSNATDPNNPLN |
| piwi | KQPTPHSRPITPSTKL-Cys |
|  | Cys-LNNKGRVGNPPPGTVV |
| tj | Cys-NKYKSSSDSLRHENEM |
|  | Cys-SQQQPTQQTQDAPPSS |
| ttk | SNEGSSSPDREDSPHSSPT-Cys |
|  | QRLRNRWSTPPSRDSEG-Cys |
| vasa | Cys-HRIGRTGRVGNRGQ |
|  | Cys-ASTDIRNQNDGFEASGG |
| VgR | DESNDCSDGSDEKNCQKTTGGK |
|  | DGDVNCEDGDDEADCEKAH |

**Table S4. Primers used in this research.**

| Name | Sequence(5'→3') | Purpose |
| --- | --- | --- |
| ds-EGFP-F | TAATACGACTCACTATAGGGAG  AGGGCGAGGGCGATGCCACCTACGG | RNAi |
| ds-EGFP-R | TAATACGACTCACTATAGGGAG  AAGTTCACCTTGATGCCGTTC | RNAi |
| ds-piwi-F | TAATACGACTCACTATAGGGAG  GAGGGTCCACTCTCTCAT | RNAi |
| ds-piwi-R | TAATACGACTCACTATAGGGAG  CTGTAGAAGTCCATTCTATTGT | RNAi |
| ds-Vgr-F | TAATACGACTCACTATAGGGAG  ATCCTCTGGAAGAAGAATAA | RNAi |
| ds-Vgr-R | TAATACGACTCACTATAGGGAG  GTATCCGTTAACATGGTG | RNAi |
| β-actin-F-615 | GAGCGAGAAATCGTTCGTGAC | qRT-PCR |
| β-actin-R-802 | GGAAGGAAGGCTGGAAGAGAG | qRT-PCR |
| eya-F-665 | CCCCAGATCCCGAGAACAAG | qRT-PCR |
| eya-R-812 | TCCATGTGGTAGCCCAACTG | qRT-PCR |
| inx2-F-734 | CGTGGCATAAATTTGGAGCG | qRT-PCR |
| inx2-R-960 | CACCTTGGTTTCGTGCTTTC | qRT-PCR |
| piwi2-F-1196 | ACAAGGGCAAGGAGATCAGC | qRT-PCR |
| piwi2-R2-1443 | GTGGACCCTCTTGTCTGGTG | qRT-PCR |
| tj-F-746 | AACGCCAGAACTACAAGGTG | qRT-PCR |
| tj-R-917 | TATGAGTTCAGTTCCGCCCT | qRT-PCR |
| ttk-F-315 | CCAACCCCTCGCCTTATTAC | qRT-PCR |
| ttk-R-515 | ATGGCACTATACTCAGCCCT | qRT-PCR |
| vasa-F-1238 | GGTTGGGGCAGCAAATAAGG | qRT-PCR |
| vasa-R-1345 | CTTGGCTATCTTCAAAGCTCCG | qRT-PCR |
| VgR-F-3431 | AAGAGATGTGCGAGGCATCC | qRT-PCR |
| VgR-R-3706 | AATTGGCAGCCATCCCTTCA | qRT-PCR |
| casp3-F-229 | ATGTCAGTGGGGAGAGATGC | qRT-PCR |
| casp3-R-345 | AGCACATGTCCGTGGTTGTA | qRT-PCR |
| casp8-F-400 | GCCATGTACTGTGTTTGTGC | qRT-PCR |
| casp8-R-632 | GGAGGACGCATAATGAAGGG | qRT-PCR |
| Bcl-2-F-328 | CGAGGTATGATGCGACGACT | qRT-PCR |
| Bcl-2-R-618 | CACTTCTGCGGGGAACTCTT | qRT-PCR |

**Data S1**

**1 Methods: Full-length RNA-seq**

**1.1. Library construction and SMRT sequencing**

Full-length RNA-Seq was performed by Gene Denovo (Guangzhou, China). RNA was individually extracted using TRIzol reagent (Invitrogen, CA, USA), and 5 mg of RNA was pooled for each library. First- and second-strand cDNA were synthesized from polyA mRNA using oligo-dT primers according to the manufacturer's instructions (Clontech SMARTER cDNA synthesis kit). Size fractionation and selection (<4 kb and >4 kb) were carried out using the BluePippin™ Size Selection System (Sage Science, Beverly, MA). The Pacific Biosciences DNA Template Prep Kit 2.0 was used for SMRTbell library construction, and the Pacific Bioscience Sequel System was used for SMRT sequencing.

**1.2 Data processing**

The raw sequencing reads of cDNA libraries were classified and clustered into transcript consensus sequences using the SMRT Link v5.0.1 pipeline supported by Pacific Biosciences. Briefly, circular consensus sequence (CCS) reads were extracted from the subread BAM files with a minimum full pass of 1 and a minimum read score of 0.65. Subsequently, the CCS reads were categorized into full-length nonchimeric (FL), nonfull-length (nFL), chimeric, and short-read sequences based on cDNA primers and the poly(A) tail signal. Reads shorter than 50 bp were discarded. The full-length nonchimeric (FLNC) reads were then clustered using iterative clustering for error (ICE) correction software to generate the cluster consensus isoforms. To enhance the accuracy of PacBio reads, two strategies were implemented. First, the non-full-length reads were used to polish the cluster consensus isoforms obtained above using Quiver software, resulting in FL-polished high-quality consensus sequences (accuracy ≥ 99%). Second, the low-quality isoforms were further corrected using Illumina short reads obtained from the same samples through the LoRDEC tool (version 0.8). Finally, the transcriptome isoform sequences were filtered by removing redundant sequences with CD-HIT-v4.6.7 software using a threshold of 0.99 identities.

**1.3 Map to the reference genome**

The corrected high-quality consensus sequences were then aligned to the reference genome (GWHALOH00000000) using GMAP. Redundant transcripts were collapsed with a minimum identity of 95% and minimum coverage of 99%. The resulting isoforms were compared to the reference genome annotation and classified into three groups: known isoforms directly annotated using the reference, novel isoforms aligned to the unannotated region of the gene, and new isoforms aligned to different exons of known isoforms.

**1.4 Functional annotation of new isoforms**

Novel and new isoforms were annotated by searching against the Nr, UniProt KB, and KOG databases using BLASTx. Function classification was performed using GO annotation, KEGG orthology, and pathway annotations. The open reading frame (ORF) of each full-length cDNA sequence was determined using ANGLE. Prediction analyses, including CPC, PLEK, Pfam-scan, and CNCI, were carried out to identify long noncoding RNAs (lncRNAs). SUPPA2 was used for alternative splicing prediction. Hmmscan was used to predict transcription factors. All the raw sequencing data were stored in the NCBI Sequence Read Archive (SRA) under the accession number “PRJNA1119192”.

**1.5 Gene structure optimization**

To optimize the gene structure in the *S.* *paramamosain* reference, each isoform was compared to the existing gene models of the GWHALOH00000000 annotation. Known isoforms were extended or shortened at the 5'UTR or 3'UTR. Other isoforms were further classified as span isoforms, exonic overlap isoforms, or intron isoforms based on their exon structure (splicing junctions).

**2 Results**

**2.1 Identifying the full-****length transcriptome of *S.paramamosain***

In this study, the full-length RNA-seq library of ovary samples from stage II and stage II *S. paramamosain* ovaries was sequenced for the first time to demonstrate the feasibility of scRNA-seq data analysis using the optimized reference genome and the full-length transcriptome of S. paramamosain and to evaluate it in crustacean samples.

A total of 37,053,370 subreads were obtained from offline data. After verifying the quantity and length of the circular consensus sequences (CCSs), 1,038,636 CCS reads were classified into four categories (Figure S6B-C), which included 924,221 FLNCs with polyA tail signals, 5′ adaptor sequences, and 3′ adapter sequences (Figure S6D). The mean length of the FLNC was 2120 bp, as shown in the Supplementary Table. Due to the greater rate of sequencing errors on the PacBio platform than on the Illumina platform, error correction was necessary. Initially, iterative clustering for error ICE correction was performed to cluster and correct the FLNC, resulting in a total of 47,775 polished high-quality isoforms (Figure S6E). These high-quality isoforms were compared to the reference genome annotation and classified into three groups: known isoforms (1395), novel isoforms (12442), and new isoforms (23701). The distributions of the 3 'and 5' UTR lengths are provided in Figures 5A and B. The use of PacBio-SMRT technology in this study significantly improved our sequencing ability and provided longer reads compared to second-generation Illumina sequencing, which laid the foundation for current research on *S.paramamosain* full-length transcripts and subsequent single-cell sequencing.

**2.2 Full-length transcript annotations**

The obtained novel and new isoforms were aligned with the KEGG, KOG, Nr, and SwissProt databases. In total, 32,466 transcripts were successfully annotated, with 21,716 transcripts found in all the databases. Specifically, the KOG, KEGG, Nr, and SwissProt annotations were assigned to 22,447, 30,545, 32,439, and 24,117 transcripts, respectively.

The distribution of the main Gene Ontology (GO) annotations is shown in Figure S7B. The most abundant subcategories of the identified biological processes were "cellular process," "single organism process," and "metabolic process." The most abundant subcategories of cellular components were "cell," "cell part," and "organelle." The most abundant subcategories of molecular functions were "catalytic activity," "binding," and "transporter activity." KEGG annotations were mostly enriched in "metabolic pathways," "protein processing in endoplasmic reticulum," and "RNA transport”.

The predicted coding sequences (CDSs) were aligned with the Animal TFDB 2.0 database using hmmscan to identify transcription factors (TFs). A total of 1251 TFs were identified and categorized into 49 families. The top ten TF families were zf-C2H2, ZBTB, MYB, CSD, TF_bZIP, homeobox, HMG, bHLH, Fork_head, and NGFIB-like (Figure S8A). These findings provide a significant foundation for future research on TFs and gene expression regulation in *S. paramamosain*.

Additionally, the analysis predicted lncRNAs and alternative splicing (AS) events. Based on the full-length transcripts that failed to align with the NR, Swiss-Prot, KEGG, and COG/KOG databases according to the CNCI and CPC approaches, a total of 3,871 lncRNAs were identified. Furthermore, a total of 2,468 AS events were identified in the transcripts of *S. paramamosain*. The proportions of the main AS events in this study were as follows: retained introns (12.58%), alternative 3' splice sites (15.97%), alternative 5' splice sites (15.89%), skipped exons (10.78%), alternative first exons (28.1%), alternative last exons (15.54%), and mutually exclusive exons (1.14%).


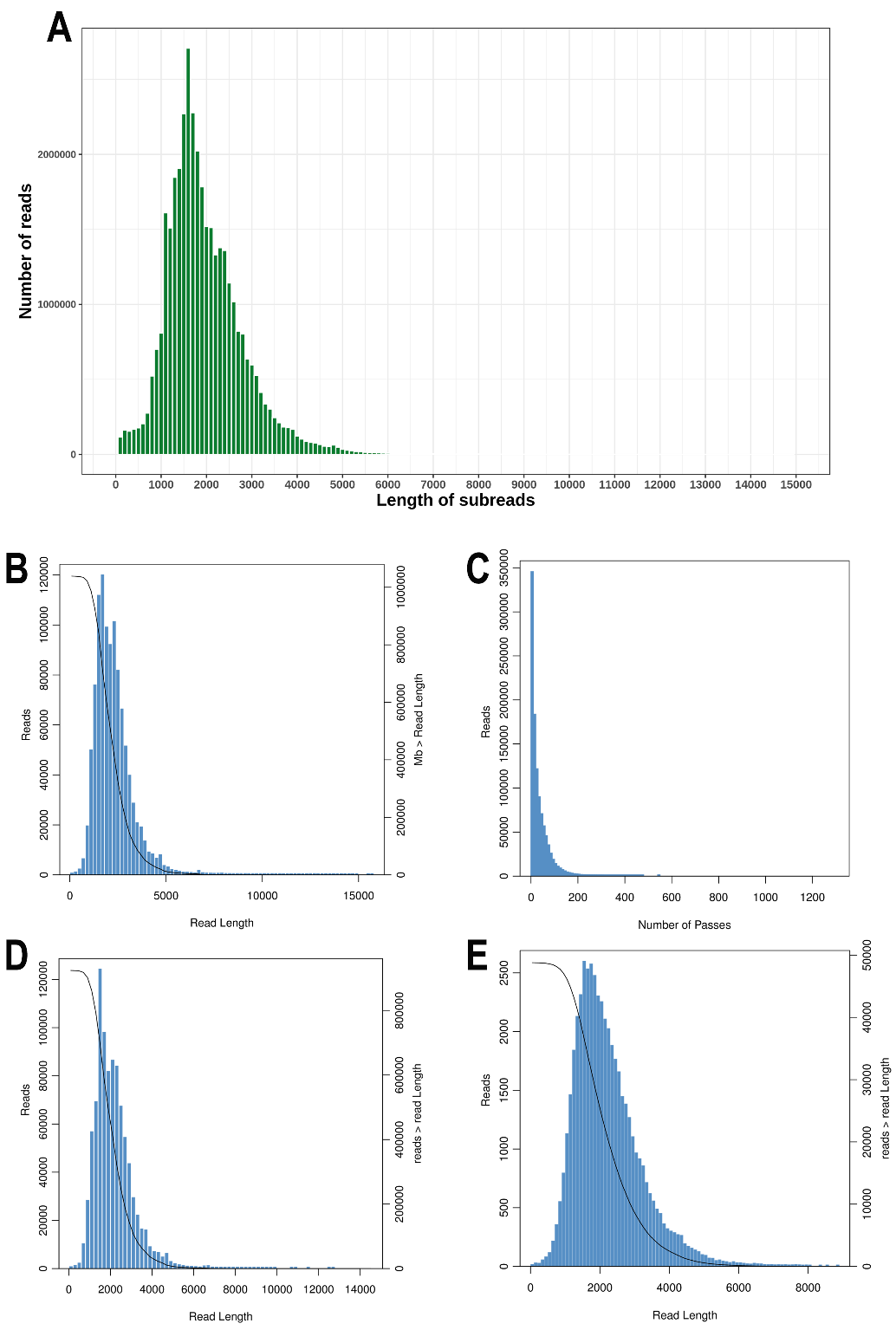


**Figure S6. Read number and length distribution after full-length transcriptomic sequencing.** (A) Subread length distribution: the abscissa represents the length of subreads, and the ordinate is the number of subreads. (B) CCS length distribution: the x-axis represents the length of the reads, and the y-axis on the left represents the coordinates of the column graph, indicating the number of reads whose length is within a certain range (x-axis); the y-axis on the right is the coordinate of the graph, indicating the number of reads whose length is greater than a certain value (x-axis). (C) CCS passes distribution: the abscissa represents the number of full passes and the ordinate represents the number of CCS sequences with corresponding full passes. (D) FLNC length distribution: the x-axis represents the length of isoforms, and the y-axis on the left represents the coordinate of the column graph, representing the number of isoforms whose length is within a certain range (x-axis); the y-axis on the right is the coordinate of the graph, indicating the number of isoforms whose length is greater than a certain value (x-axis). (E) Consistent sequence length distribution: the abscissa represents the length of the consistent sequence, the left ordinate represents the number of sequences with the length, and the right ordinate represents the number of sequences with the length greater than a certain value (x-axis).


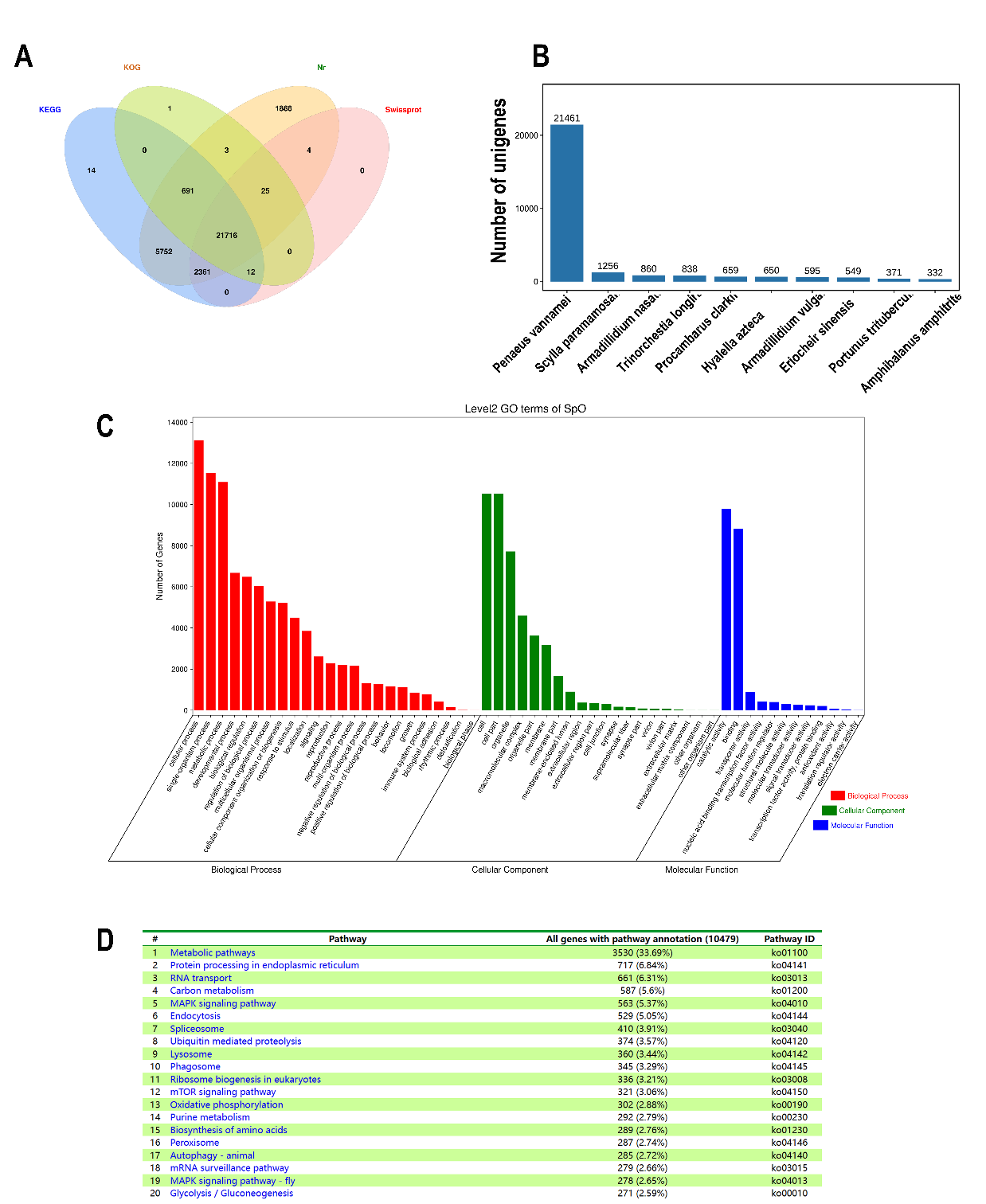


**Figure S7. Functional annotations of the full-length transcripts with Nr, SwissProt, GO and KEGG.** (A) Venn analysis of annotation results of four databases: Nr, Swiss Prot, KEGG and COG/KOG. (B) Statistical map of species distribution (only the top 10 species are shown): after comparing isoform sequences with the Nr database by BlastX, the sequence with the best (lowest E value) hit to the isoform in the Nr database was taken as the corresponding homologous sequence to determine homologous sequences of the species. The number of homologous sequences of each species was statistically compared. (C) Go function classification chart. (D) Distributions of the KEGG pathways.


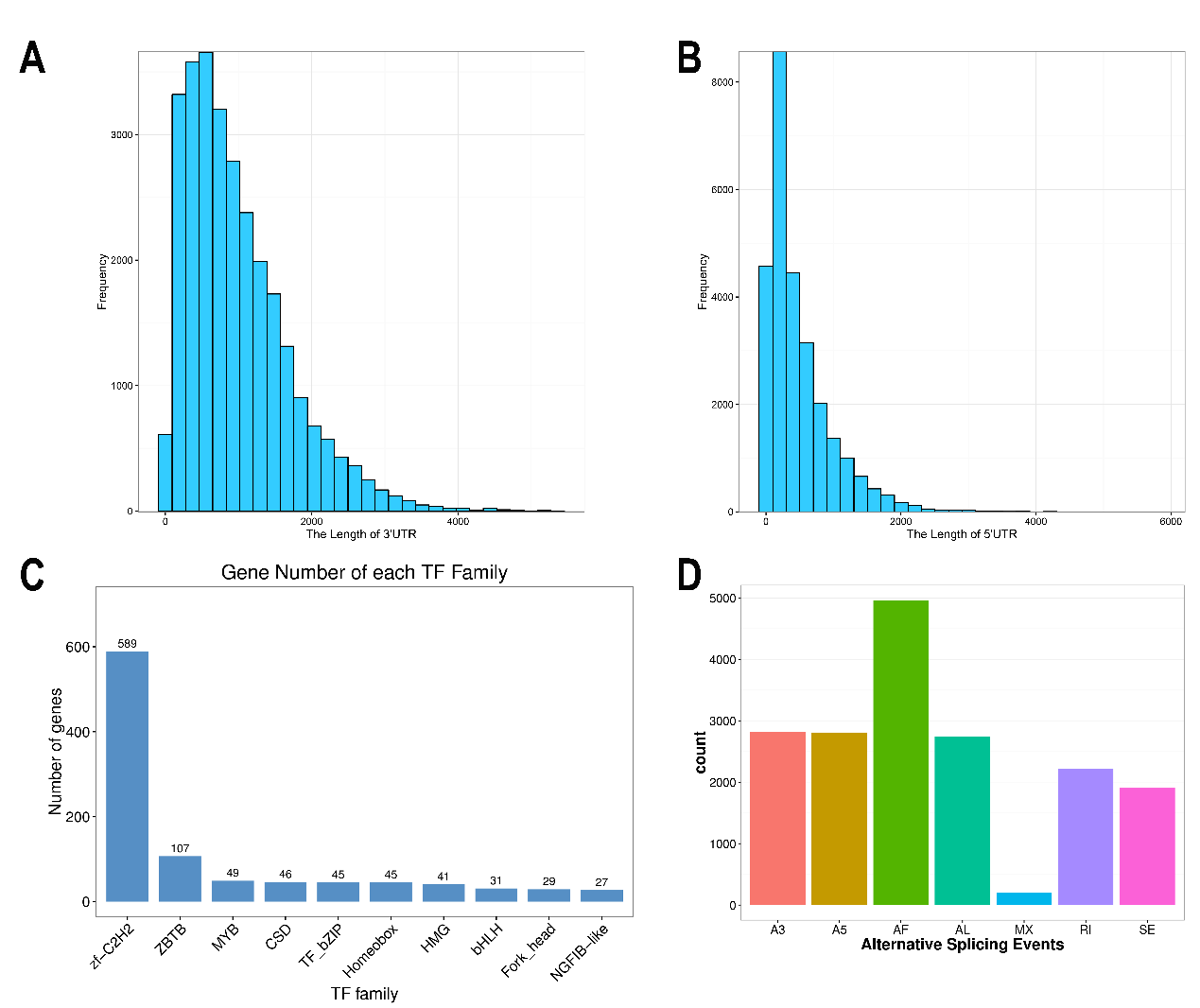


**Figure S8. Prediction of the coding sequences and transcription factors.** (A) The length of 3’ UTR. (B) The length of 5’ UTR. (C) TF family distribution (top 10). (D) The main AS events.
